# Italian Adaptation and Psychometric Properties of the Prejudice Against Immigrants Scale (PAIS): Assessment of Validity, Reliability, and Measure Invariance

**DOI:** 10.3389/fpsyg.2020.01797

**Published:** 2020-07-28

**Authors:** Marco Salvati, Emanuele Basili, Nicola Carone, Mauro Giacomantonio

**Affiliations:** ^1^Department of Social and Developmental Psychology, Sapienza University, Rome, Italy; ^2^Department of Psychology, Sapienza University, Rome, Italy; ^3^Department of Brain and Behavioral Sciences, University of Pavia, Pavia, Italy

**Keywords:** immigrants, prejudice, PAIS, psychometric properties, scale adaptation

## Abstract

The aim of the current study was to adapt and validate the Prejudice Against Immigrants Scale (PAIS) in the Italian context, based on the Prejudice Against Asylum Seekers Scale by Anderson (2018). The validity, reliability, and measurement invariance across gender, age, and educational levels of the scale were assessed through three sources, which involved 306 Italian individuals (*N*_men_ = 151, 49.3%) between 18 and 60 years old. Both exploratory and confirmatory factor analyses (CFA) confirmed the two-factor solution of the original instrument by excluding two items, which were present in the previous validation study. The first factor is *classical prejudice against immigrants*, which maps onto theoretical derivations of classical and old-fashioned prejudices, whereas the second factor is *conditional prejudice against immigrants*, which maps onto theoretical derivations of subtle and modern prejudices. Findings of the multigroup CFAs demonstrated full configural and metric invariance and partial scalar invariance of the scale across gender, age, and educational level. The analyses confirmed that PAIS has high levels of reliability and criterion and construct validity, showing findings that are comparable to those of Anderson (2018). These results suggest that PAIS presents very good psychometric properties and could be considered a valid and reliable instrument to measure prejudice against immigrants, by enabling Italian researchers to detect both covert and more subtle forms of prejudice against immigrants. Limitations and further directions are discussed.

## Introduction

Immigration has been a relevant social issue throughout history and remains so today. A recent survey launched by the European Commission (2018) found that approximately 37 million people born outside Europe reside in Europe, accounting for approximately 7% of its total population, and further increases of migration are expected. Such influx of immigrants to Europe has intensified the debate over the acceptance of newcomers into European countries, the changing fabric of European societies, and the coexistence-related challenges posed by immigrants to members of the host society (Stephan et al., 2005). In this vein, immigration has been recently seen as the most important issue facing the European Union, cited by more than a third (39%) of respondents (Standard Eurobarometer 86, 2017). This being the case, it is intuitively evident that immigration and integration represent two politically sensitive issues across Europe, particularly due to the increased number of immigrant arrivals over the last few years (European Commission, 2018).

Several reasons may motivate immigration, including the changing distribution of employment opportunities, population imbalances, natural disasters, and the actions of the nation states themselves (Ceobanu and Escandell, 2010). Although immigration often benefits both immigrants and the host nation, feelings that threaten social order, social cohesion, and traditions and norms, as well as prejudice and discrimination often accompany the phenomena (Stephan et al., 2005; Pettigrew et al., 2010; Rustenbach, 2010; Pettigrew, 2013; Pistella et al., 2020; Salvati et al., 2019a). This comes along with the tendency of members of the host country to overestimate the size of the immigrant population, which, in turn, results in more negative attitudes against them (Schneider, 2008; Gorodzeisky and Semyonov, 2019). Insofar as evidence indicates that hostility and discrimination toward immigrants have negative economic, political, and social effects on both the host country and the immigrant group (Stephan et al., 1999), researchers have attempted to understand the causes of prejudice against immigrants. This would help both address European citizens’ concerns about the ability of their countries to manage immigration-related challenges and develop effective policies for enhancing integration and preventing intergroup conflicts (Green et al., 2019).

The literature suggests that both individual-level and country-level factors may affect prejudice and negative attitudes toward immigrants (Davidov et al., 2019). At the individual level, studies focusing on the sociodemographic correlates of prejudice against immigrants found that people who are socioeconomically disadvantaged or earn low income are unemployed, less educated, and with high levels of religiosity, as well as those who support political conservatism are more likely to display greater prejudice (Hello et al., 2002; O’Rourke and Sinnott, 2006; Carvacho et al., 2013; Turoy-Smith et al., 2013; Anderson and Ferguson, 2018; Piumatti and Russo, 2019).

In terms of psychological aspects associated with greater prejudice against immigrants, relevant factors include social dominance orientation (SDO) – which describes individuals’ acceptance of group-based status hierarchy, where some groups are more advantaged than others (Pratto et al., 1994) – and a right-wing authoritarianism (RWA) – which characterizes individuals’ beliefs about the need for submission and obedience to authority, conformity to traditional norms and values, and aggression toward those who fail or refuse to submit or conform to that authority (Asbrock et al., 2010). Both are stable ideological orientations that characterize people’s general views about status hierarchies and intergroup relations. A further relevant factor for prejudice is the need for cognitive closure (NfC), which describes an individual’s search for beliefs that they can know with certainty (Roets and Van Hiel, 2011a). Because immigrants are agents of change, members of the host country with a high NfC are more likely to have negative attitudes toward immigrants because they represent change that prevents them from having secure knowledge (Baldner and Pierro, 2019).

At the country level, explanations of prejudice and negative attitudes toward immigrants include size of the immigrant population and economic conditions (Semyonov et al., 2006; Meuleman et al., 2009; Kuntz et al., 2017), media coverage of immigration (Vaes et al., 2015; Schlueter et al., 2019), or country integration policies (Schlueter et al., 2013, 2019; Green et al., 2019). It is suggested that large or increasing immigration flows, perception of poorer economic conditions, policies that do not promote integration of immigrants, and/or negative media reports related to immigration may all result in more negative attitudes toward immigration in a country.

Because of its structural societal attributes, Italy is a country of particular interest for studying prejudice against immigrants. First, as of January 2018, the number of foreign nationals residing in Italy comprised 8.4% of the country’s population, with an increase of 17,972 over the previous year (ISTAT, 2018). The figure includes children born in Italy to foreign nationals but excludes both foreign nationals who have acquired Italian nationality and illegal migrants, whose numbers are difficult to determine. Second, in Italy, immigrants are subject to various degrees of discrimination in various contexts, such as workplace and housing markets (Ambrosini, 2013a; Piumatti and Russo, 2019). Third, several political parties’ and ideological forces’ key principles aim specifically to prevent migration and oppose promotion of immigrant-inclusive social policies (Ambrosini, 2013b). Such a scenario is also likely amplified by the media (Vaes et al., 2015), resulting in a distorted perception of the percentage of immigrants actually present in Italy compared to the total population. In this vein, a recent survey launched by the European Commission (2018) found that 73% of the Italian citizens overestimate the presence of immigrants in the country: Italian citizens in fact believe that immigrants represent 25% of the total population residing in Italy, although the exact figure is less than 10%. This is the highest gap in comparison to all other European countries.

### The Current Research and Hypotheses

The present study adapted and validated the Prejudice Against Immigrants Scale (PAIS) in the Italian context, based on the Prejudice Against Asylum Seekers Scale (PAAS) by Anderson (2018). Therefore, the validity, reliability, and measurement invariance (MI) across gender, age, and educational levels of the scale were assessed. Following Anderson (2018), two conceptual components of attitudes toward immigrants were defined, and items were generated that could capture these components. The first component is *classical prejudice against immigrants* (PAIS-CL), which can be defined as the deliberate and unconcealed reporting of blatant prejudice against immigrants (e.g., “Immigrants are a waste of time, money, and space”). These attitudes map on to theoretical derivations of classical and old-fashioned prejudices. The second component is *conditional prejudice against immigrants* (PAIS-CO), which is a form of modern prejudice defined as negative attitudes that are socially acceptable to express because they are endorsed concurrently with a qualifying statement (e.g., “Immigrants can enter our country as long as they abide our laws”). These attitudes map on to theoretical derivations of subtle and modern prejudices. In summary, the PAIS scale was designed with two subscales that comprised eight items, each in order to measure old and new forms of prejudice against immigrants. The psychometric properties of the PAIS were tested through the following hypotheses:

Hypothesis 1: *Factor structure of the scale* – We expect to replicate and confirm the two-factor structure of the 16-item PAAS by Anderson (2018), in the Italian context, after replacing the term “asylum seekers” with “immigrants.” Specifically, in exploratory factor analysis (EFA), we expect two distinct, yet interrelated, factors to emerge, whereas in the confirmatory factor analysis (CFA), we expect to confirm the structure found in the original instrument validation study (Anderson, 2018). The first dimension should be the PAIS-CL, which refers to the deliberate and unconcealed blatant prejudice against immigrants. The second dimension should be the PAIS-CO, which indicates a form of modern prejudice, which refers to negative attitudes, more socially acceptable.
Hypothesis 2: *Gender, Age, and Education Invariance* – We expect to verify the MI of the instruments for several groups of participants. Specifically, we expect that the two-factor structure will be solid and confirmed for both male and female participants, individuals younger than 26 and older than 26 years, and people with a high and a low educational level (individuals with a bachelor’s degree at least and individuals with at most a diploma, respectively).
Hypothesis 3: *Reliability* – As done in Anderson’s original validation study (2018), we expect to find very good internal consistency estimates for both dimensions. Specifically, we hypothesize that Cronbach α of both PAIS-CL and PAIS-CO would be higher than 0.70.
Hypothesis 4: Validity –

a) Criterion validity: We hypothesize that the PAIS would correlate with existing measures of racism, such as the scale by Pettigrew and Meertens (1995) validated in Italy by Leone et al. (2006). Furthermore, to confirm the theoretical distinction between the PAIS-CL and PAIS-CO dimensions, the correlation should be stronger for PAIS-CL than for PAIS-CO, as found by Anderson (2018).
b) Construct validity: We expect to confirm the relationships found in previous studies between PAIS dimensions and ideological and dispositional variables such as religiosity (Anderson, 2018; Piumatti and Russo, 2019), political orientation (Greenhalgh and Watt, 2015), political interest (Wylie and Forest, 1992), SDO (Cohrs and Asbrock, 2009), RWA (Asbrock et al., 2010), and NfC (Roets and Van Hiel, 2011a). Also, we explored the relationship sociodemographics (Carvacho et al., 2013; Anderson and Ferguson, 2018; Salvati et al., 2019a), Specifically, based on existing literature, male, older, and less-educated individuals would be more likely to report high prejudice against immigrants, than female, younger, and more-educated individuals. Furthermore, higher scores on PAIS dimensions are expected to be associated with conservative political orientation, lower political interest, higher religiosity, higher SDO, higher RWA, and higher NfC. Finally, we expect that correlations would be stronger for PAIS-CL than for PAIS-CO (Anderson, 2018).

## Materials and Methods

### Participants and Procedure

The data of the current research were derived from several sources. First, the authors collected part of the data from a convenience sample using personal networks and asking participants to respond to an online survey (*n* = 95). Second, the PAIS was included in the online questionnaires of other two concurrent studies investigating other psychological topics (i.e., sexual prejudice against sexual minority people), unrelated to prejudice against immigrants (*n* = 116 and *n* = 95, respectively). Thus, the total sample consisted in 306 Italian individuals between 18 and 60 years (mean = 26.19, *SD* = 7.07). All the demographics and descriptives are reported in Table 1. Before participating in the surveys, all participants read and signed their consent form. No compensation was provided for participating in the studies. After completing the surveys, participants were thanked and debriefed.

**TABLE 1:** Descriptive (means, standard deviations, frequencies, and percentages) of the sample’s characteristics, divided by gender.

**Descriptive of the sample’s characteristics**	**Participants**		
	**Total Sample *N* = 306 (100%)**	**Men *N* = 151 (49.3%)**	**Women *N* = 155 (50.7%)**
Age	26.19 (7.07)	27.58 (7.28)	24.84 (6.61)
**Education level**			
Middle School Diploma	13 (4.2%)	10 (6.6%)	3 (1.9%)
High School Diploma	157 (51.3%)	74 (49.0%)	83 (53.5%)
Bachelor’s degree	62 (20.3%)	31 (20.5%)	31 (20.0%)
Master’s degree	57 (18.6%)	28 (18.5%)	29 (18.7%)
Postgraduate level	17 (5.6%)	8 (5.3%)	9 (5.8%)
PAMS-CL	2.14 (1.17)	2.43 (1.23)	1.86 (1.03)
PAMS-CO	3.80 (1.35)	4.11 (1.27)	3.49 (1.36)

	**Total sample *N* = 190 (100%)**	**Men *N* = 122 (64.2%)**	**Women *N* = 68 (35.8%)**

Political orientation	3.37 (1.36)	3.52 (1.42)	3.09 (1.22)
Political interest	2.65 (0.96)	2.67 (0.97)	2.63 (0.94)
Religiosity	2.78 (1.08)	2.80 (1.12)	2.74 (1.01)
BCC	3.13 (0.60)	3.17 (0.54)	3.04 (0.68)

	**Total sample *N* = 95 (100%)**	**Men *N* = 27 (28.4%)**	**Women *N* = 68 (71.6%)**

SDO	2.16 (0.95)	2.38 (0.97)	2.07 (0.93)
RWA	2.81 (1.13)	3.01 (1.30)	2.73 (1.06)
Racism	2.78 (0.72)	3.00 (0.62)	2.69 (0.74)

### Measures

#### Demographics

Demographics were asked in all the three surveys of the current research (Table 1). Participants were asked to indicate their gender (1 = men, 2 = women), age, and educational level (1 = middle school diploma, 2 = high school diploma, 3 = bachelor’s degree, 4 = master’s degree, 5 = postgraduate level). To test age and educational invariance of the measure, two groups were created, respectively: participants younger than 26 years (*n* = 159) and older than 26 years (*n* = 147); participants without a degree (*n* = 170) and with at least a degree (*n* = 136).

#### Prejudice Against Immigrants Scale

A team of three academic experts on immigration issues worked on the Italian translation and adaptation of the 16 original items of the PAAS (Anderson, 2018). For the purpose of the research, we replaced the term “asylum seekers” with “immigrants” for a more inclusive use of the instrument. Furthermore, the more generic term “immigrants” is the one most commonly used in the Italian language to refer to people arriving in Italy from developing countries (especially from Africa), regardless of whether they are asylum seekers, or regular or irregular, or already have a resident permit and have been living in Italy for years. Subsequently, the Italian items were sent to a native English speaker who worked on the back-translation to verify the adequacy of the translation. The original scale consisted in 16 items divided into two dimensions with eight items each: classic prejudice (e.g., “Immigrants need to go back to where they came from”) and conditional prejudice (e.g., “Immigrants might struggle to integrate into our system”). Items asked participants for their degree of agreement on a Likert scale ranging from 1 = not at all, to 7 = completely. All items can be read in Table 2.

**TABLE 2:** Factor loadings of the items of the three factors resulting from EFA (*N* = 147).

**Items**	**Factor 1**	**Factor 2**
1. Gli immigrati devono tornarsene da dove sono venuti.	0.91	
2. Gli immigrati sono una Perdita di tempo, denaro e spazio.	0.90	
4. Gli immigrati creano più problemi che altro.	0.82	
7. Gli immigrati non-dovrebbero essere un nostro problema.	0.66	
8. Gli immigrati non-sono I benvenuti nel nostro Paese.	0.62	
10. Gli immigrati fingono soltanto di aver bisogno di aiuto.	0.67	
13. Gli immigrati non-sono in grado di integrarsi nella nostra società	0.55	
14. Gli immigrati sono troppo pericolosi per averli nel nostro Paese	0.86	
3. Gli immigrati hanno bisogno di aiuto, tuttavia ci sono persone nel nostro Paese che ne hanno più bisogno.	0.39	0.44
5. Gli immigrati dovrebbero ritornare nei loro Paesi d’origine, una volta tornati sicuri.	0.31	0.57
6. Gli immigrati dovrebbero poter venire qui solo se non-hanno una storia criminale alle spalle		0.43
9. Gli immigrati vanno bene, fintantoché non ne prendiamo troppi	0.34	0.50
11. Gli immigrati potrebbero avere difficoltà ad integrarsi nel nostro sistema		0.61
12. Gli immigrati avrebbero maggiori probabilità di adattarsi se sapessero parlare l’Italiano		0.59
15. Gli immigrati dovrebbero avere ammessi nel nostro Paese, ma dopo i migranti regolari		0.59
16. Gli immigrati possono entrare nel nostro Paese fintantoché rispettano le nostre leggi		0.70

*All factor loadings < | 0.30| were omitted. Factor 1 was named “Classic Preudice against Immigrants (PAIS-CL)”; Factor 2 was named “Conditionl Prejudice against Immigrants (PAIS-CO)”; Factor 3 was named “Positive Beliefs toward Immigrants.”*

#### Political Orientation and Political Interest

Participants were asked to indicate their political orientation on a seven-point scale ranging from 1 = *extremely progressive* to 7 = *extremely conservative*. Subsequently three items asked: “How important is politics for you?”; “How much are you interested in politics?”; “How politically committed are you?” Participants responded on a five-point Likert scale, ranging from 1 = not at all, to 5 = completely. A total score of political interest was computed by averaging the three items.

#### Religiosity

Four items investigated participants’ religiosity asking the following questions on five-point Likert scale, ranging from 1 = not at all, to 5 = completely: “How important is religion for you?”; “Have you received a religious education?”; “Currently, I consider myself a religious person”; “I try to stick to the precepts of my religion.” A total score of religiosity was computed by averaging the four items.

#### Need for Closure

We administered the brief 15-item version of the Need for Closure Scale (Roets and Van Hiel, 2011b) to measure participants’ NfC. People with high NfC prefer order and structure in their lives; prefer predictability, desiring secure and stable knowledge; experience an urgent desire to reach swift decisions; feel discomfort with ambiguity; and are closed-minded to alternative opinions (Webster and Kruglanski, 1994). Participants responded on a five-point Likert scale ranging from 1 = completely disagree to 5 = completely agree, and higher scores corresponded to a higher need for closure. Examples items were “I don’t like situations that are uncertain” or “I feel irritated when one person disagrees with what everyone else in a group believes.”

#### Social Dominance Orientation

Participants completed a shortened six-item SDO scale (Pratto et al., 1994), already used in previous studies about attitudes toward human and non-human outgroups (Dhont et al., 2014). Social dominance orientation refers to the fundamental desire to achieve and maintain group-based dominance and inequality among social groups, and it was found to be a predictor of intergroup attitudes, including racial and ethnic prejudice (Hodson and Esses, 2005; Cohrs and Asbrock, 2009). Participants responded on a seven-point Likert scale, ranging from 1 = completely disagree to 7 = completely agree and higher scores on the scale corresponded to higher SDO. Example items were “Some groups of people are just more worthy than others” and “Superior groups should dominate inferior groups.”

#### Right-Wing Authoritarianism

Right-wing authoritarianism refers to an uncritical subjection to authority, feeling of aggression toward norm violators, and strict adherence to conventional norms and values (Aiello et al., 2004), and it was found to be a strong predictor of racial and ethnic prejudice (Ekehammar et al., 2004; Asbrock et al., 2010). We selected the best six items from the 14-item short version of the Right-Wing Authoritarianism Scale (Rattazzi et al., 2007), and participants responded on a seven-point Likert scale, ranging from 1 = completely disagree to 7 = completely agree. Higher scores corresponded to higher RWA, and example items were “What our country really needs instead of more ‘civil rights’ is a good stiff dose of law and order” and “Everyone should have their own lifestyle, religious beliefs, and sexual preferences, even if it makes them different from everyone else.”

#### Racism

We measure participants’ blatant and subtle racism by the Italian adaptation of the scale by Pettigrew and Meertens (1995), developed by Leone et al. (2006). Despite the original intentions of the authors, however, they failed in demonstrating the empirical evidence for the two dimensions of the scale and suggest to researchers a monodimensional use of the instrument, following indications by Coenders et al. (2001). Thus, we computed a total racism score on the 16 items as suggested by Coenders et al. (2001). Participants responded on a six-point Likert scale from 1 = completely disagree to 6 = completely agree. Example items were “It would not be a problem for me to work in the employ of a suitably qualified immigrant” and “Immigrants living in our country transmit to their children values and skills that are not those necessary to succeed in Italy.”

### Analytic Plan

Descriptive analyses and correlations were conducted using the Statistical Package for the Social Science (SPSS, v. 23), whereas Mplus v.7 (Muthén and Muthén, 2012) was used to conduct EFAs and CFAs. First, the factorial structure of the PAIS was examined through EFA. The total sample was randomly divided into two samples of similar size, using the SPSS random split routine to select approximately 50% of study participants for each group. Random sample I (*n* = 147) was used to conduct an EFA, and data from the second split sample (*n* = 159) were used to conduct a CFA. Through this methodology, the first sample can be used to develop a good fitting solution, and the final model is then fitted in the second sample to determine its replicability with independent data. Based on the indications from the EFAs, we then tested the factorial validity of the PAIS scale through CFA using all the items from the original scale and dropping items that were found problematic in the confirmatory phases. In all the models, maximum likelihood estimator was used, and we relied on common fit indices to evaluate model to data fit: χ*^2^*, comparative fit index (CFI), root mean square error of approximation (RMSEA), and standardized root mean squared residual (SRMR), expecting values of CFI > 0.90, RMSEA < 0.08, and SRMR < 0.06 (Hu and Bentler, 1999; Kline, 1999).

Subsequently, in order to test for MI across genders, ages, and education, we conducted several multigroup CFAs (MGCFA) to determine whether the PAIS factors were equivalent at a *configural* (same factorial structure across groups), *metric* (factor loadings are equivalent for corresponding items across groups), and *scalar* (intercepts are equivalent across groups) invariance levels across genders, ages, and education (Vandenberg and Lance, 2000). Nested models with incremental constrains were implemented, and differences between models were computed using a χ^2^-test, which is frequently used. In case of significant difference in the fit indices, we relied on the highest MIs to identify which parameter needed to be freely estimated (Brown, 2015) in order to test for partial invariance. Finally, once full or partial scalar MI was established, latent means comparisons could be tested in order to examine whether there were significantly different scale’s factor means between groups. To make such comparison, one group was chosen as a reference group by fixing its latent means to zero, whereas factor means of the other group were freely estimated (Vandenberg and Lance, 2000; Brown, 2015). Finally, criterion validity and construct validity were tested through correlations on all the data whose measures were available.

## Results

### Preliminary Analyses

Preliminary analyses were conducted to test normality and multicollinearity of our measures. Skewness and kurtosis values were computed to test normality, whereas correlations were run to test multicollinearity among the measures. Specifically, on the one hand, the following thresholds were defined for skewness and kurtosis, respectively: lower than 3 and lower than 8 (Kline, 1999; Salvati et al., 2019b). On the other hand, a maximum value of |0.80| was considered an indicator of absence of multicollinearity (Field, 2009).

The results showed that neither normality nor multicollinearity was an issue. Indeed, all the skewness values ranged from | 0.02| to | 1.70|, and all the kurtosis values ranged from | 0.01| to | 1.76|, except for age, whose kurtosis value was 4.01, but lower than the defined threshold. Finally, as expected, no correlation exceeded the value of | 0.80|, confirming the absence of multicollinearity. All skewness, kurtosis, and correlation values are reported in Table 3.

**TABLE 3:** Correlations, means, standard deviations, kurtosis, skewness, and Cronbach’s alpha values.

	**1**	**2**	**3**	**4**	**5**	**6**	**7**	**8**	**9**	**10**	**11**	**12**
1. PAIS-CL	1											
2. PAIS-CO	0.66**	1										
3. Gender	−0.25**	−0.23**	1									
4. Age	0.07	–0.05	−0.19**	1								
5. Education	−0.17**	–0.09	0.03	0.31**	1							
6. Political orientation	0.57**	0.47**	−0.15*	–0.04	–0.12	1						
7. Political interest	–0.13	−0.15*	–0.02	–0.07	0.05	−0.22**	1					
8. Religiosity	0.24**	0.31**	–0.03	0.10	–0.04	0.22**	0.07	1				
9. SDO	0.37**	0.26**	–0.15	0.05	–0.14	0.30**	−0.28**	0.04	1			
10. RWA	0.52**	0.42**	–0.11	0.14	–0.17	0.51**	−0.34**	0.45**	0.38**	1		
11. NfC	0.38**	0.32**	–0.11	0.06	−0.16*	0.27**	–0.09	0.25**	0.15	0.32**	1	
12. Racism	0.80**	0.62**	–0.19	0.14	−0.26*	0.51**	−0.40**	0.36**	0.43**	0.66**	0.47**	1
N	306	306	306	306	306	190	190	190	95	95	190	95
Mean	2.14	3.80	–	26.19	–	3.37	2.65	2.78	2.16	2.81	3.12	2.78
Standard deviation	1.17	1.35	–	7.07	–	1.36	0.96	1.08	0.95	1.13	0.60	0.72
Skewness	1.38	0.09	–	1.70	–	0.29	0.15	0.34	0.72	0.51	0.02	0.70
Kurtosis	1.76	–0.72	–	4.01	–	–0.48	–0.84	–0.89	–0.21	–0.64	0.19	–0.01
Cronbach’s alpha	0.91	0.82	–	–	–	–	0.84	0.86	0.62	0.67	0.81	0.82

*p < 0.05, **p < 0.01.

### Exploratory Factor Analysis

The factorial structure of the PAIS was examined through EFA on the 16 items of the Italian translation. Oblique rotation with the Geomin procedure was used. Model fits and factor loadings for the one-, two-, and three-factors solutions showed that the two-factor solution best represented the data [χ^2^_(__89__)_ = 158.869, *p* < 0.001; CFI = 0.94, Tucker Lewis Index (TLI) = 0.93; SRMR = 0.041; RMSEA = 0.073, 90% confidence interval (CI) = 0.054 –0.091]. The eigenvalues were 7.15, 2.08, and 0.09. Factor 1 (classic prejudice) included items 1, 2, 4, 7, 8, 10, 13, and 14; factor 2 (conditional prejudice) included items 3, 5, 6, 9, 11, 12, 15, and 16. As shown in Table 2, item 3 was the only item presenting very similar loadings on both factors. However, after the rotation, the loading on the expected factor (conditional prejudice) was higher and significant (*p* < 0.05) that the loading on factor 1 and the correlation between item 3 and factor 1 was higher than the correlation with factor 2 (*r* = 0.65 vs. *r* = 0.58, respectively). Accordingly, the item was retained for the subsequent test of CFA. The factor loadings (all > 0.40) confirmed the hypothesized structure of the PAIS Italian version, which is comparable to that of the original questionnaire (Anderson, 2018). Specifically, supporting Hypothesis 1, the EFA revealed the two expected underlying structures of the total scale, and the items factored onto their hypothesized factors with all the items providing initial support for the factor structure hypothesis.

### Confirmatory Factor Analysis

In order to test the two-factor model suggested by the EFA, we tested a CFA on the second subsample (N2). Initially, the fit provided by the first model was not entirely satisfactory [χ^2^_(__103__)_ = 233.831, *p* < 0.001; CFI = 0.90; RMSEA = 0.089, 90% CI = 0.74-0.105; SRMR = 0.072]; therefore, we proceeded to inspect factor loadings and modification indices. We noted that items 11, “Immigrants might struggle to integrate into our system,” and 12, “Immigrants are more likely to fit in if they can speak Italian,” from the conditional prejudice factor had very low loadings (i.e., λ = 0.11 and λ = 0.19, respectively), and we decided to remove them from the model. The new model showed a significant improvement in the model fit when removing both items 11 and 12 [Δχ^2^_(__27__)_ = 88.583, *p* < 0.001]. Furthermore, according to modification indices’ suggestions, we included a residual correlation between items 1 (“Immigrants need to go back to where they came from”) and 2 (“Immigrants are a waste of time, money and space”) from classic prejudice factor. The final model confirmed the measurement model hypothesized for the PAIS: the two-factor model had a good fit with the data [χ^2^_(__75__)_ = 131.833, *p* < 0.001; CFI = 0.95; RMSEA = 0.069, 90% CI = 0.049-0.088; SRMR = 0.049], the standardized loadings ranged from 0.41 to 0.89, and the two factors were positively correlated (*r* = 0.84, *p* < 0.001).

### Measurement Invariance Across Gender, Age, and Education

We used the factorial structure derived from the CFA to examine MI across participants’ gender, age, and education, specifically a series of nested MGCFA models with increasing parameter constraints to test for configural, metric, and scalar invariance. Model’s fit statistics are reported in Table 4.

**TABLE 4:** Tests for measurement invariance of PAIS measurement model across gender, age, and education: summary of goodness-of-fit statistics.

	**χ^2^**	***df***	**CFI**	**TLI**	**RMSEA**	**SRMR**	**Model comparison**	**χ^2^_diff_**	**Δ*df***
**Measurement invariance across gender**									
Male (*n* = 151)	144.792*	75	0.936	0.922	0.079 (0.059–0.098)	0.057			
Female (*n* = 155)	157.382*	75	0.936	0.922	0.084 (0.066–0.103)	0.056			
Model 1. configural invariance	311.786*	152	0.933	0.919	0.083 (0.070–0.096)	0.077			
Model 2. metric invariance	326.519*	163	0.931	0.923	0.081 (0.068–0.094)	0.072	2 vs. 1	14.733	11
Model 3. scalar invariance	358.362*	175	0.923	0.920	0.083 (0.070–0.095)	0.079	3 vs. 2	31.843*	12
Model 4. partial scalar invariance^a^	346.075*	174	0.928	0.924	0.080 (0.068–0.093)	0.076	4 vs. 2	19.556	11
**Measurement invariance across age**									
Under 26 (*n* = 147)	112.913*	75	0.966	0.958	0.059 (0.035–0.080)	0.052			
Over 26 (*n* = 159)	165.937*	75	0.933	0.919	0.087 (0.069–0.105)	0.059			
Model 1. configural invariance	292.522*	152	0.942	0.932	0.078 (0.064–0.091)	0.085			
Model 2. metric invariance	295.761*	163	0.946	0.940	0.073 (0.060–0.086)	0.065	2 vs. 1	3.23	11
Model 3. scalar invariance	323.296*	175	0.940	0.937	0.074 (0.062–0.087)	0.068	3 vs. 2	27.535*	12
Model 4. partial scalar invariance^b^	310.139*	174	0.945	0.942	0.072 (0.058–0.084)	0.066	4 vs. 2	14.378	11
**Measurement invariance across education**									
Low education group (*n* = 170)	146.737*	75	0.948	0.937	0.075 (0.057–0.093)				
High education group (*n* = 136)	118.767*	75	0.957	0.947	0.066 (0.042–0.087)	0.048			
Model 1. configural invariance	274.003*	152	0.949	0.939	0.072 (0.059–0.086)	0.068			
Model 2. metric invariance	292.581*	163	0.946	0.940	0.072 (0.059–0.085)	0.068	2 vs. 1	18.578	11
Model 3. scalar invariance	318.487*	175	0.940	0.938	0.073 (0.060–0.086)	0.069	3 vs. 2	25.906*	12
Model 4. partial scalar invariance^c^	305.671*	174	0.945	0.943	0.070 (0.057–0.083)	0.069	4 vs. 2	13.09	11

χ *^2^, Chi-square Goodness of Fit; df, degrees of freedom; CFI, Comparative Fit Index; TLI, Tucker–Lewis index; RMSEA, Root Mean Square Error of Approximation. Upper and lower limits for the observed RMSEA confidence intervals (RMSEA CI); SRMR, Standardized Root Mean Squared Residual. All the Δ index comparisons are made with respect to the previous model. ^∗^ p = 0.01. ^*a*^Free intercept of Conditional Prejudice: item 5 (“Gli immigrati non-sono i benvenuti nel nostro Paese”). ^*b*^Free intercept of Conditional Prejudice: item 6 (“Gli immigrati fingono soltanto di aver bisogno di aiuto”). Free intercept of Conditional Prejudice: item 6 (“Gli immigrati fingono soltanto di aver bisogno di aiuto”).*

Regarding gender, full configural and metric invariance were found, showing that the scale’s structure and factor loadings were equivalent between men and women. The test of scalar model showed a significantly worse fit compared to the metric model [Δχ^2^_(__12__)_ = 31.843, *p* < 0.001] showing that full scalar invariance was not supported. We then tested for partial scalar invariance. Through the examination of the modification indices, we identified which item intercepts needed to be freely estimated, and we proceeded to free item 5 intercept (“Immigrants should return to their country once safe to do so,” conditional prejudice factor). The final model showed acceptable fit to the data [χ^2^_(__174__)_ = 346.075, *p* < 0.001; CFI = 0.93; TLI = 0.92; RMSEA = 0.080, 90% CI = 0.068-0.093; SRMR = 0.076], supporting partial scalar invariance and showing that items intercepts were comparable and equivalent between men and women.

In order to test MI for age, participants were divided in two age groups comprising those participants under and over 26 years of age. As for MI regarding participants’ gender, the multigroup models showed full configural and metric invariance, while full scalar invariance was not supported, [Δχ^2^_(__12__)_ = 27.535, *p* < 0.001]. Modification indices suggested to free item 6 intercept (“Immigrants should only come here if they don’t have a criminal history,” conditional prejudice factor), and the resulting model showed acceptable fit to the data [χ^2^_(__174__)_ = 310.139, *p* < 0.001; CFI = 0.940; TLI = 0.94; RMSEA = 0.072, 90% CI = 0.058-0.084; SRMR = 0.066], supporting partial scalar invariance and showing that scale’s factor structure, factor loadings, and intercepts were equivalent between younger and older participants.

In order to test MI for education, participants were divided in two groups comprising those participants who completed fewer years of education (“low education group,” e.g., middle and secondary school degree) and those who reported a higher level of education (“high education group,” e.g., university degree). The multigroup models showed full configural and metric invariance, whereas full scalar invariance was not supported [Δχ^2^_(__12__)_ = 25.906, *p* < 0.05]. We then tested for partial scalar invariance, and modification indices suggested we free the intercept of item 6 (“Immigrants should only come here if they don’t have a criminal history,” conditional prejudice factor), and the resulting model showed good fit to the data [χ^2^_(__174__)_ = 305.671, *p* < 0.001; CFI = 0.94; TLI = 0.94; RMSEA = 0.070, 90% CI = 0.057–0.083; SRMR = 0.069], showing the scale’s factor structure, factor loadings, and intercepts were equivalent between those who had a higher or lower education.

Finally, once scalar invariance was found, we were able to test whether latent means were significantly different between groups. Regarding gender, latent means comparisons showed that women scored significantly lower than men in both classic prejudice (*z* = −4.26, *p* < 0.001) and conditional prejudice (*z* = −4.39, *p* < 0.001). Regarding age, older participants scored significantly lower than younger participants in their mean levels of classical prejudice (*z* = −2.11, *p* < 0.001).

Finally, regarding participants’ education, latent means comparisons showed that participants with higher education scored significantly lower than those with lower education in their mean levels of conditional prejudice (*z* = −2.25, *p* < 0.05).

### Reliability

To test the reliability of the two dimensions of PAIS, we computed internal consistency estimates through Cronbach α. As expected, the values for both the classical and conditional prejudice against immigrants were higher than 0.70, indicating an optimal internal consistency. Specifically, Cronbach α’s were 0.91 for PAIS-CL and 0.82 for PAIS-CO. These findings are perfectly comparable to the values found in the several samples by Anderson’s validation study (2018). Indeed, in the four samples of his research, Cronbach α’s for PAIS-CL ranged from 0.86 to 0.93, whereas Cronbach α’s for PAIS-CO ranged from 0.79 to 0.89. Cronbach α was computed for all the other measures used in this research, and they can be found in Table 2.

### Criterion Validity

Criterion validity was tested by running correlations between the two dimensions of PAIS and an existing measure of racism that was originally developed to measure blatant and subtle racism separately (Pettigrew and Meertens, 1995). However, several authors demonstrated that the instrument does not have the empirical support for the two dimensions, and for this reason, they suggested the use of a total racism score (Coenders et al., 2001; Leone et al., 2006). Thus, we chose to use the total score of the scale and compute the associations with PAIS-CL and PAIS-CO, respectively. Confirming our hypothesis, both the dimensions were significantly related with the measure of racism. Furthermore, as expected, the association of racism with PAIS-CL, *r* = 0.80, *p* < 0.01, was stronger in magnitude rather than with PAIS-CO, *r* = 0.62, *p* < 0.01, confirming the same pattern of results found in the validation study by Anderson (2018) and contributing to support the theoretical distinction between PAIS-CL and PAIS-CO.

### Construct Validity

Construct validity was verified by running correlations among the two dimensions of PAIS and several sociodemographic, ideological, and dispositional variables found to be associated with ethnic prejudice in previous literature. Regarding the relationships with sociodemographic, both PAIS-CL and PAIS-CO were equally and moderately associated to gender (*r* = −0.25, *p* < 0.01; *r* = −0.23, *p* < 0.01, respectively) and not related to age, whereas PAIS-CL and not PAIS-CO showed a negative weak relationship with education (*r* = −0.17, *p* < 0.01). Specifically, male participants, compared to female participants, were more likely to report both classical and conditional prejudice against immigrants, whereas individuals with a lower level of education, compared to individuals with a higher level of education, were more likely to report higher classical, but not conditional prejudice.

Observing the correlations with politic and religious variables, the results indicated that a conservative political orientation and a high religiosity were more likely to be associated with both classical (*r* = 0.57, *p* < 0.01; *r* = 0.24, *p* < 0.01, respectively) and conditional prejudice against immigrants (*r* = 0.47, *p* < 0.01; *r* = 0.31, *p* < 0.0.1, respectively), rather than a progressive political orientation and a low religiosity. Conversely, the political interest was weakly associated only with PAIS-CO (*r* = −0.15, *p* < 0.05), but not with PAIS-CL.

Regarding the associations with dispositional and ideological variables, the results showed that SDO, RWA, and NfC were positively and moderately associated to both classical and conditional prejudice against immigrants. As expected, the three correlations with PAIS-CL were slightly stronger than correlations with PAIS-CO, confirming the results found by the original validation study of the instrument (Anderson, 2018). Specifically, participants with high levels of SDO, RWA, and NfC were more likely to report higher classical and conditional prejudice against immigrants, compared to participants with low levels of SDO, RWA, and NfC.

## Discussion

Interest in both understanding prejudice against social minority groups and developing effective policies for enhancing integration and preventing intergroup conflicts has resulted in the need to develop measures that can accurately assess multidimensional aspects (i.e., classical and conditional) of attitudes toward immigrants. To this aim, the current study evaluated the psychometric properties of the PAIS in the Italian context, which is characterized by quite hostile attitudes toward immigrants (Salvati et al., 2019a) and where the distorted perception of the percentage of immigrants actually present in Italy led to the highest gap in comparison to all other European countries (European Commission, 2018).

The findings presented in this article suggest that PAIS is a valid and reliable measure of prejudice against immigrants in Italy. First, the factor structure of the scale was hypothesized to comprise two subscales that would measure classical and conditional prejudices. Moreover, it was predicted that these subscales would be distinct, yet correlated. This was explored in EFA and confirmed in CFA. Specifically, our results from CFAs supported the two-factor solution by excluding two items from the *conditional prejudice* factor, which were present in the prior validation study (Anderson, 2018). The two excluded items were removed because they demonstrated low factor loadings in CFA. This might be due to the translation process. Indeed, on the one hand, item 11 explicitly refers to “our system,” an expression that could be biased because of the cultural differences between Italian and Australian systems. On the other hand, item 12 refers to the possibility that immigrants would be more likely to fit if they can speak “Italian” (instead of “English” as in the original version of the scale). The low factor loading of such item could be interpreted by considering that Italian participants might have retained very unlikely that immigrants could know Italian, compared to English for Australians. However, confirmation of the two-factor solution provided additional support for the theoretically formulated factors of the PAIS.

Second, we tested whether MI could be established across participants’ age, gender, and levels of education. Findings of the MGCFAs demonstrate full configural, metric invariance, and partial scalar invariance. First, the establishment of full configural and metric invariance indicates that the same items are associated with the same latent factors and that factor loadings for all items are consistent between younger and older participants, men and women, and those who have higher or lower educational levels. Regarding scalar invariance, we found partial MI for the three variables. Specifically, only item 5 (“Immigrants should return to their country once safe to do so”) was non-invariant for gender, and only item 6 (“Immigrants should only come here if they don’t have a criminal history”) was non-invariant for both age and educational level.

According to Chen (2007), intercept non-invariance might occur when one group displays a propensity to answer more strongly to an item even though it shows the same factor mean of the other group(s). However, when the number of non-invariant items is small compared to the total, the latent factor means to test group differences should not be drastically impacted (Millsap and Kwok, 2004; Sass, 2011). In this particular case, the reason why males and females showed a different initial mean only on the item that states “immigrants should return to their country once safe to do so” is not so evident, as well as the reasons why there are age and educational level mean differences only on the item that states that immigrants should only come in Italy if they do not have a criminal history. Perhaps, the content of these items reflects an aspect of threat to which female, younger, and higher-educated people might be more sensitive, compared to men and older and lower-educated individuals (Salvati et al., 2019a).

Subsequently, we compared latent group means between samples and found several mean differences between cultural groups. Specifically, women scored significantly lower than did men in both classic prejudice and conditional prejudice. Such a difference is not surprising in light of previous literature showing that men are likely to report more negative attitudes toward immigrants compared to women (Turoy-Smith et al., 2013; Anderson, 2018; Piumatti and Russo, 2019). Regarding age, older participants scored significantly lower than did younger participants in their mean levels of classical prejudice. This result could be read considering the lack of robustness and consistency of relationships found in literature between age and negative attitudes toward immigrants (Anderson and Ferguson, 2018; Salvati et al., 2019a). Finally, regarding participants’ education, latent means comparisons showed that participants with higher education scored significantly lower than did those with lower education in their mean levels of conditional prejudice. This result is in line with previous literature showing that less-educated individuals are more likely to display negative attitudes toward immigrants compared to more-educated people (O’Rourke and Sinnott, 2006; Carvacho et al., 2013).

Third, the findings indicate that this scale has high levels of reliability as demonstrated by estimated internal consistency coefficients above the suggested cutoff (>0.70). Finally, three forms of validation evidence were conducted. First, criterion validity was demonstrated by strong correlations with the unidimensional measure of racism, with people reporting higher levels of racism also reporting higher levels of prejudice against immigrants. Second, construct validity was demonstrated by correlations with SDO, RWA, and NfC, which are well-known dispositional variables related to prejudice (Pratto et al., 1994; Asbrock et al., 2010; Roets and Van Hiel, 2011a; Baldner and Pierro, 2019). Finally, known-groups validity was demonstrated by empirically based sociodemographic variable predictions, with males and participants reporting lower education, conservative political orientation, and higher religiosity showing greater prejudice against immigrants. Importantly, such findings are comparable to those of Anderson (2018).

Several limitations must be considered when interpreting the findings. First, as noted by Anderson (2018), items reflecting conditional prejudice (i.e., statements that allow negative attitudes to be expressed because they are endorsed concurrently with a qualifying statement) lend themselves to present multiple issues within the one item. This can undermine the clarity of what a participant’s response might mean. Future revisions of this scale, along with the possibility to also qualitatively collect respondents’ interpretations of such items through open-ended questions, could help address this issue. Second, correlation between the two factors of PAIS shown in CFA analysis is quite impressive. Unfortunately, the original validation of the instrument (Anderson, 2018) does not report this datum. For this, it was not possible to compare our result with that one of the original scale. Thus, such an aspect should be kept in mind for the future use of the instrument.

Third, the convenience nature of the sample might impact the generalizability of the scale. In this vein, because data were collected from multiple sources, not all related to prejudice against immigrants, the chance that the other study variables might have influenced participants’ responses cannot be ruled out. Fourth, the PAIS was administered during a time period in which immigration was a particularly sensitive topic in the Italian context, given that most political propaganda was based on this subject. Therefore, it may be possible that this condition might have led respondents to polarize their answers. Finally, it is worth noting that immigrants represent an intersectional group who include characteristics of several stigmatized minorities social, religious, and ethnic minorities (e.g., asylum seekers, Muslims, Romanians). Whether such intersectionality had an additive or interactive effect on attitudes toward the target was not investigated.

Notwithstanding these limitations, the present study offers preliminary indications that the PAIS is a valid and reliable instrument to measure prejudice against immigrants, which enables Italian researchers to detect both covert and more subtle forms of prejudice. This issue is particularly relevant because there is broad agreement that prejudice may be expressed through different attributions and under various circumstances (e.g., Akrami et al., 2000; Anderson, 2018). Overall, the findings further encourage researchers to translate and validate the PAIS in diverse sociocultural contexts.

## Data Availability Statement

The raw data supporting the conclusions of this article will be made available by the authors, without undue reservation.

## Ethics Statement

The studies involving human participants were reviewed and approved by the Ethical Committee of the Department of Social and Developmental Psychology, Sapienza University, Rome. The patients/participants provided their written informed consent to participate in this study.

## Author Contributions

MS contributed to the design and execute the study, contributed to conduct data analyses and wrote the manuscript. EB contributed to conduct data analyses, and wrote the manuscript. NC contributed to write the manuscript and to edit the final manuscript. MG contributed to the design and execute the study and edited the final manuscript. All authors contributed to the article and approved the submitted version.

## Conflict of Interest

The authors declare that the research was conducted in the absence of any commercial or financial relationships that could be construed as a potential conflict of interest.
